# RAB39B: A novel biomarker for acute myeloid leukemia identified via multi-omics and functional validation

**DOI:** 10.1515/med-2025-1168

**Published:** 2025-03-28

**Authors:** Shuo Chen, Mengxing Li, Jishi Wang

**Affiliations:** Department of Hematology, Affiliated Medicine of Guizhou Medical University, Guiyang, 550001, China; Graduate School, Guizhou Medical University, Guiyang, 550001, China; Hematology Laboratory, Guizhou Medical University, Guiyang, 550001, China

**Keywords:** RAB39B, immune infiltration, m6A modification, apoptosis, prognosis, acute myeloid leukemia

## Abstract

**Background:**

The objective of this research was to investigate the involvement of RAB39B in acute myeloid leukemia (AML) using bioinformatics analysis and *in vitro* experiments for validation.

**Methods:**

In this article, RNA sequencing data from The Cancer Genome Atlas and genotype-tissue expression were utilized to analyze the expression of RAB39BA and identify differentially expressed genes.

**Results:**

AML exhibited elevated expression of RAB39B in diverse tumor types. In laboratory experiments, it has been demonstrated that RAB39B exhibits a significant expression level in AML cell lines when compared to normal peripheral blood monocytes. Moreover, RAB39B is closely linked to the growth and programmed cell death of AML cells.

**Conclusion:**

In conclusion, RAB39B shows potential as a biomarker for the identification and prediction of AML, contributing to the growth and cell death processes in AML.

## 1 Introduction

AML, also known as acute myeloid leukemia, is a cancerous tumor characterized by its significant diversity, unpredictable outlook, and elevated fatality rate. At present, the primary factors that determine risk stratification and treatment options primarily consist of cytogenetic and molecular abnormalities [1,2]. Gene mutations occurring during the processes of cell proliferation, differentiation, and apoptosis serve as the foundation for the development of AML. Nevertheless, the development of AML is highly intricate and has yet to be precisely understood. The current targeted drugs or conventional combination chemotherapy are still not effective enough for treating AML, with the overall survival (OS) rate for AML patients still hovering at a low level, below 30% at the 5-year survival rate [3]. A significant contributor to this poor prognosis is the profound heterogeneity exhibited by AML in terms of its biological characteristics and clinical manifestations [4].

Over the past decade, extensive research has unraveled a lot of genetic mutations, chromosomal abnormalities, and gene expression patterns in AML [5]. These studies have highlighted the profound impact of specific genetic alterations on patient outcomes. Mutations in NPM1 or CEBPA are frequently observed in AML and are generally indicative of a favorable prognosis, potentially slowing disease progression by modulating processes such as cellular differentiation and apoptosis [6,7]. However, it is important to note that not all AML patients harbor these mutations. Moreover, even among those who do, there can be significant variability in clinical courses and treatment outcomes. Conversely, mutations in genes such as FLT3, IDH1/IDH2, RUNX1, and TP53 are not only associated with distinct AML subtypes but also correlate with adverse prognosis and influence patient responsiveness to therapy [7,8,9,10]. Despite the remarkable advancements in AML biomarker research, current cytogenetic and molecular markers still fall short in fully elucidating the complex biological underpinnings and clinical heterogeneity of this disease. Hence, the discovery and recognition of novel biomarkers can enhance our comprehension of the molecular processes involved in AML development and facilitate the creation of personalized diagnostic and therapeutic strategies. This, in turn, could significantly contribute to the management, prognostic categorization, and advancement of targeted medications for AML [11,12].

Rab proteins, small GTPases, regulate the movement of vesicles in the cells of eukaryotic organisms. Rab39B belongs to the Rab protein family. The expression of Rab39B can be observed in different tissues of humans. It is situated on the Xq28 chromosome and is composed of two exons that cover a length of 3764 base pairs in the genomic DNA of humans [13]. The primary emphasis of previous research on Rab39B has been on its involvement in neurodevelopmental abnormalities, such as Parkinson’s disease, cognitive impairment, and autism spectrum disorder [14,15]. According to reports, cancer researchers have found a high expression of Rab39B in germ cell tumors, gastric stromal tumors, and diffuse large B-cell lymphomas [16,17,18]. Additional research has indicated that Rab39B is linked to the tumor microenvironment and the outlook of pancreatic adenocarcinoma [19]. Furthermore, RAB39B is believed to be involved in regulating the PI3K/Akt/mTOR signaling pathway to affect cell behavior [20]. Apoptosis and autophagy pathways have been linked to Rab39B in recent research [21,22]. Emerging evidence also highlights Rab39B’s potential role in intracellular vesicle trafficking and signaling pathways related to cancer cell proliferation, migration, and invasion, suggesting it may represent a novel therapeutic target [16,23,24]. Inhibiting Rab39B’s function or expression could disrupt these crucial processes, offering a new thought for AML treatment [16]. Nevertheless, the precise molecular mechanisms linked to Rab39B remain undisclosed, particularly in relation to mechanisms related to tumors. Until now, there has been a lack of research on the molecular mechanism, biological role, and future outlook of Rab39B in AML.

Through the examination of information obtained from accessible databases, we assessed the variation in RAB39B expression in both pan-cancer and AML. To comprehend the potential roles of RAB39B in AML, we employed protein‒protein interactions (PPIs), coexpressed genes, immune infiltration, m6A-related genes, cuproptosis-related genes, and ceRNA networks. Furthermore, we employed multidimensional analysis to assess the autonomous predictive significance of RAB39B in AML. Additionally, we performed *in vitro* assays to confirm the distinct manifestation of RAB39B and assess its impact on the growth and programmed cell death of AML cells.

So far, research on the expression, prognosis, and mechanism of RAB39B in AML is still blank. Our study provides the possibility for RAB39B as a new biomarker for AML and establishes sufficient scientific evidence for clinical decision-making and risk management.

## 2 Materials and methods

### 2.1 Analysis of biological information and sources of data

The Cancer Genome Atlas (TCGA) and genotype-tissue expression (GTEx) pan-cancer RNA-seq data were uniformly processed from UCSC XENA (https://xenabrowser.net/datapages/). The data of AML samples from HTSeq FPKM and HTSeq Count were acquired from TCGA platform, whereas the RNA-seq data from GTEx, encompassing normal tissue samples, were utilized to facilitate a comparative analysis of RAB39B expression levels between AML and normal samples for subsequent analysis [25,26].

### 2.2 LinkedOmics analysis

The LinkedOmics database, accessible at http://www.linkedomics.org/login.php, houses a comprehensive collection of multiomics and clinical data derived from 32 different cancer types as part of TCGA project [27]. Using the Link Finder module of Linked Omics, we rigorously conducted a search for differentially expressed genes (DEGs) that are associated with RAB39B in AML (*n* = 150). The Pearson correlation coefficient was utilized to perform statistical analysis on genes coexpressed with RAB39B, and the corresponding outcomes were presented using volcano plots and heatmaps. The LinkInterpreter module was utilized to perform Gene Ontology (GO) analysis and Kyoto Encyclopedia of Genes and Genomes (KEGG) pathway enrichment analysis to annotate genes that were coexpressed with RAB39B.

### 2.3 Search Tool for the Retrieval of Interacting Genes (STRING) analysis

The purpose of STRING (https://cn.string-db.org/) was to combine all identified and anticipated connections among proteins, encompassing both physical interactions and functional associations [28]. Using the STRING database, we performed an analysis and built a PPI network for RAB39B in this research.

### 2.4 Construction of the ceRNA network

starBase is a freely available platform that systematically investigates RNA and protein RNA interaction networks through the analysis of 37 CLIP-seq datasets, including PAR CLIP, HITS CLIP, iCLIP, and CLASH [29]. starBase was utilized to predict the prospective miRNA of RAB39B. Screen for shared miRNAs predicted by PITA, miRmap, and TargetScan. Furthermore, we examined the association between the expression of these potential medications and RAB39B to identify miRNAs that are better suited for ceRNA circumstances.

MiRNet2.0 (www.mirnet.ca/miRNet/home.xhtml) is a web-based tool that provides a comprehensive and interactive analysis platform for exploring miRNA‒target interactions. Visit the homepage of ca/miRNet.xhtml) is a network visualization analysis platform centered on miRNA [30]. Using miRNet2.0, we built a correlation network to predict the target lncRNAs for miR-152-3p and miR-582-5p. We also screened lncRNAs that had a more significant correlation with miRNAs. Ultimately, the ceRNA network for DLBCL was constructed by utilizing the negative correlation between the expression levels of miRNA mRNA and miRNA lncRNA, with the key lncRNA miRNA mRNA (RAB39B) being included.

### 2.5 Analysis of immune infiltration

Conduct immune infiltration analysis utilizing single sample gene set enrichment analysis (ssGSEA) for single sample gene set enrichment analysis. The immune infiltration status of the corresponding cloud data was calculated using the markers of 24 immune cells provided in the Immunity article [31], utilizing the ssGSEA algorithm from the GSVA package [1.46.0] of R (4.2.1). Investigation of genes associated with cuproptosis and genes related to m6A methylation. The RNAseq data (level 3) and corresponding clinical information of AML tumors were acquired from TCGA dataset available at https//portal.gdc.com. Subsequently, the R software package pheatmap was utilized to construct the multigene correlation map. We examined the correlation between RAB39B and m6A-associated genes, along with genes related to cuproptosis [32,33]. Spearman’s correlation analysis was employed to depict the association among numerical variables lacking a normal distribution, and *P* values below 0.05 were deemed statistically significant.

### 2.6 Prognostic correlation analysis

The pROC package was utilized for receiver operating characteristic (ROC) analysis of the data, and the diagnostic ROC curve was then visualized using ggplot2. To investigate the prognostic significance of RAB39B in AML, Kaplan‒Meier curves were generated using GEPIA2 (http//gepia2.cancer-pku.cn/) [34]. Proportional hazards hypothesis testing and Cox regression analysis were conducted using the survival package. Additionally, the rms package was utilized to create nomogram-related models, visualize them, and generate nomograms and calibration plots. A calibration curve can be evaluated graphically by mapping the predicted probability of the nomogram to the observed rate, with the 45° line indicating the best value predicted.

### 2.7 Cell culture

OCI-AML-3, KG-1, MOLM-13, and MV4-11, which are human AML cell lines, were acquired from Cell Biotechnology (Shanghai) Co., Ltd. and cultured following the suggested procedure. According to previous research [35], AML cells were transfected with siRNA (sense 3′-UCAUUCUUCAGAAGAGGUUTT-5′; antisense 3′-AACCUCUUCUGAAGAAUGATT-3000′).

### 2.8 Quantitative polymerase chain reaction performed in real time

Quantitative PCR (qPCR) was used to analyze the expression of the AML cell lines OCI-AML-3, KG-1, MOLM-13, and MV4-11. The RAB39B primers used were 5′-CTGGGATACAGCGGGTCAAG-3′ (forward primer) and 5′-GAAGGACCTGCGGTTGGTAA-3′ (reverse primer) [35].

### 2.9 Western blot

The collected cells were mixed with lysis buffer and protein inhibitor, followed by lysis on ice for 30 min. Subsequently, centrifugation at a speed of 12,000 rpm was performed for 15 min. The liquid above the sediment was collected, and a specific quantity of 5 times concentrated sample buffer was introduced. The specimen was subjected to a temperature of 100°C for a duration of 10 min. The protein concentration was measured utilizing a Denovix ultra micro ultraviolet‒visible spectrophotometer. The protein gel was transferred onto a PVDF membrane, followed by blocking with 5% skim milk for a duration of 2 h. Subsequently, the membrane was incubated overnight with the primary antibody (Catalog Number.12162-1-AP, Proteintech) at 4°C. The membrane was exposed to the secondary antibody (ZB2301, Beijing Zhongshan) at ambient temperature for a duration of 60 min. Photographs were taken using a Tanon fully automatic chemiluminescence imaging system, and the grayscale values were read using the system’s analysis software. The trial was conducted three times.

### 2.10 Cell proliferation detection

Cells were placed in 96-well plates and incubated for the necessary duration to conduct the cell proliferation assay. The experimental group was treated with CCK-8 reagent (Beijing Suo Laibao Technology Co., Ltd.) by adding it to each well. The treated wells were then placed in a 37°C, 5% CO_2_ incubator and incubated for a duration of 2 h. A microplate reader was used to measure the OD value of the solution at a wavelength of 450 nm.

### 2.11 Flow cytometry

To analyze apoptosis, we utilized a kit from Hangzhou Lianke Biotechnology Co., Ltd., to detect cell apoptosis.

The cells were rinsed with PBS. Then, trypsin (without EDTA) was introduced for cell digestion. Next, the culture medium was added to halt the reaction and prevent excessive digestion. After that, centrifugation was performed to eliminate the supernatant. The cells were washed with cold PBS, and the cell concentration was adjusted to 1 × 10^6^/mL. Centrifuge at 1,500 rpm for 5 min and discard the supernatant. Next, 500 µL of 1× binding buffer working solution was added. Gently resuspend the cells and introduce 5 µL of ANNEXIN V-FITC and 10 µL of PI. Prepare separate staining tubes and a blank tube for the cells. The blank tube should not contain any staining agent, while the two single staining tubes should have 5 µL of ANNEXIN V-FITC and 10 µL of PI. Following a gentle swirling motion, it was allowed to sit in a dimly lit area at ambient temperature for a duration of 5 min.

The Beckman FC500 flow cytometry instrument was utilized to load the device, calibrate the voltage of the empty tube, adjust the compensation of the tube with single staining, configure the parameters, and upload the sample tube for data analysis.

### 2.12 Statistical analysis

R (4.2.1) was used for the analysis and visualization of all statistical data and graphics. The chi-square test, Fisher’s exact test, Kruskal‒Wallis test, and Wilcoxon signed-rank test were utilized for the analysis of clinical information. Statistically significant results were defined as *P* values <0.05 in all analyses.

## 3 Results

### 3.1 Expression of RAB39B in both pan-cancer and AML

**Figure 1:**
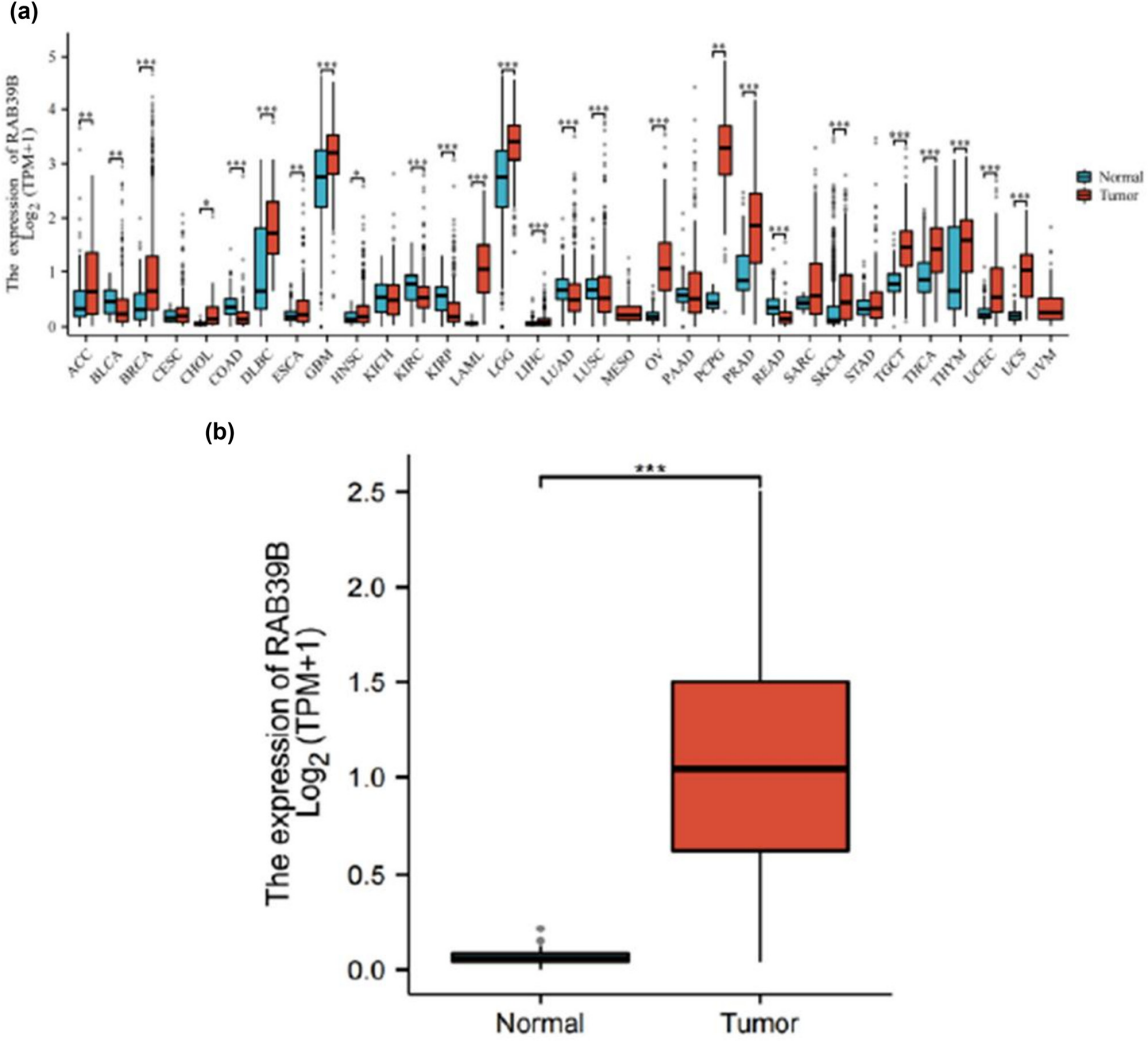
AML samples exhibited greater expression of RAB39B than normal samples. (a) Comparison of the expression levels of RAB39B in both normal and pan-cancer samples. (b) Comparison of RAB39B expression levels in normal and AML samples. A comparison of the two groups was conducted using the Wilcoxon rank sum test. NS: *P* = 0.05 or higher; *P < 0.05; **P < 0.01; ***P < 0.001.

The information obtained from UCSC XENA undergoes a standardized Tour process, where it is transformed into RNAseq data in the TPM format of TCGA and GTEx. A notable variation in RAB39 expression was observed among 26 cancer types (Figure 1a), including AML (LAML), when comparing the expression of RAB39B in normal and tumor samples from TCGA and GTEx databases (Figure 1b).

### 3.2 RAB39B PPI network and co-expression analysis in AML

**Figure 2:**
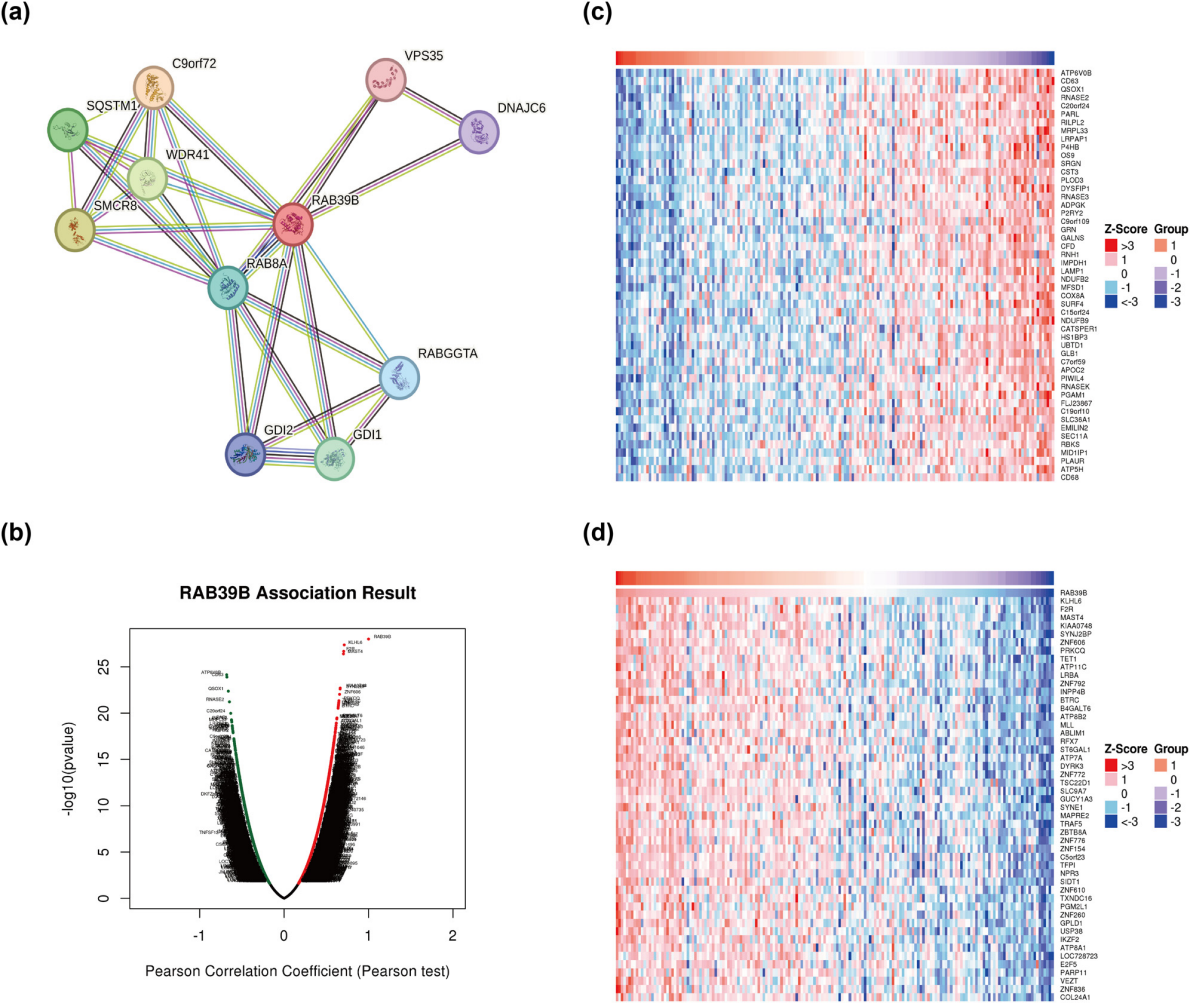
Analysis of genes associated with RAB39B and their enrichment. (a) RAB39B’s PPI network. (b) AML coexpressed genes were analyzed by LinkedOmics and visualized by Volcano plots. (c) The top 50 genes that have a positive correlation with RAB39B in AML. (d) The 50 most negatively correlated genes with RAB39B in AML.

By utilizing the STRING database, we set a medium confidence score threshold of >0.400 to construct a PPI network for RAB39B (Figure 2a), resulting in the identification of a total of 10 genes associated with RAB39B. The analysis indicated that RAB39B was linked to WDR41, a complex that possesses GEF activity and controls autophagy. This complex includes SMCR8, C9orf72, RAB8A, RABGGTA, GDI1, GDI2, SQSTM1, VPS35, and DNAJC6. RAB8A belongs to the Ras oncogene family among these correlated proteins, whereas SMCR8, C9orf72, WDR41, and SQSTM1 are linked to autophagy.

**Figure 3:**
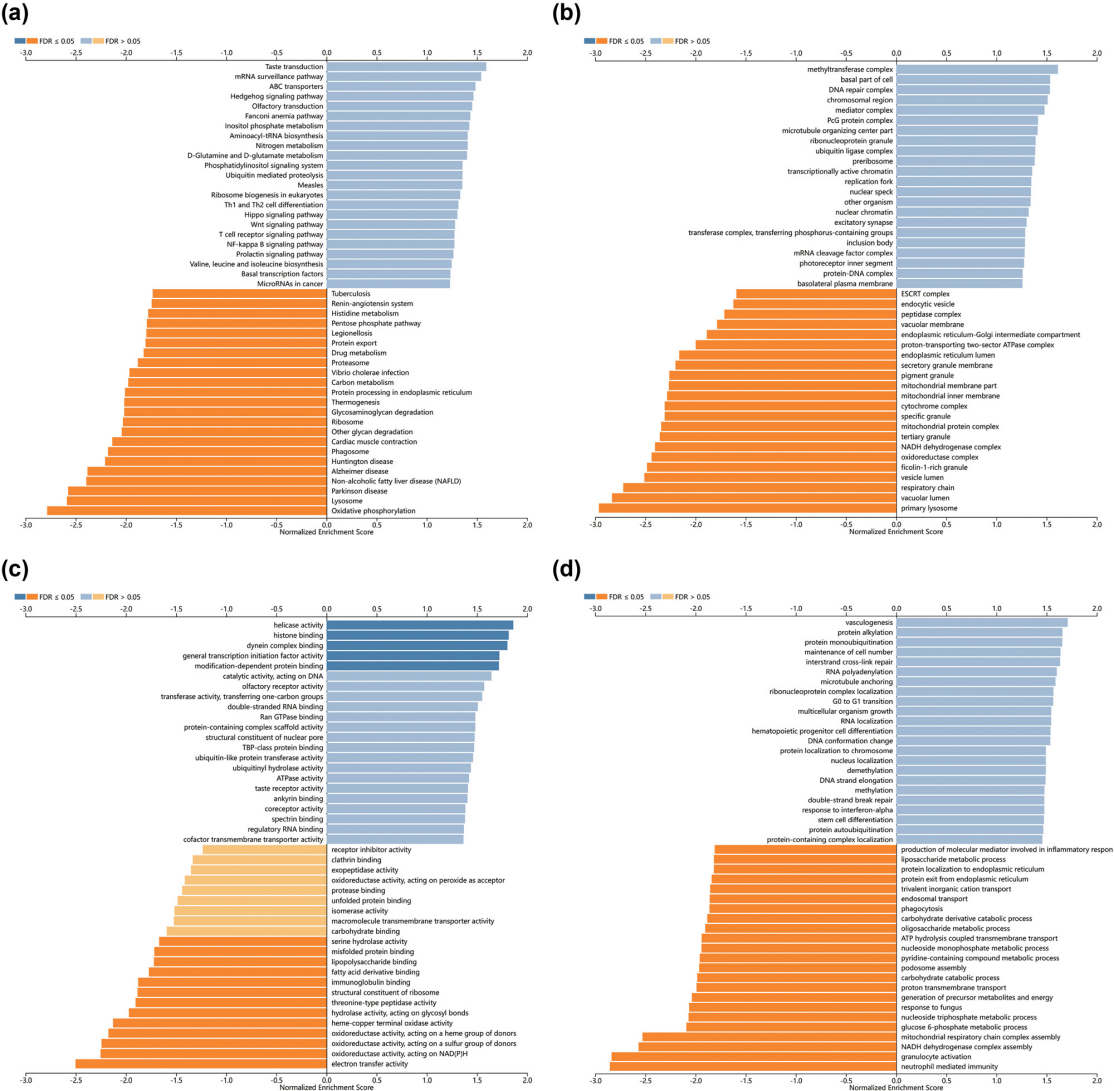
Enrichment analysis of RAB39B coexpressed genes in AML. GO biological process (a), cellular component (b), and molecular function (c) analyses were used to annotate coexpressed genes of RAB39B. (d) KEGG pathway analysis of RAB39B coexpressed genes.

To gain a deeper understanding of the biological role of RAB39B, we examined the coexpression of RAB39B in AML through the utilization of the LinkedOmics database. The analysis reveals that a total of 5,710 genes (dark red dots) exhibit a positive correlation with RAB39B, whereas 3,712 genes (dark green dots) demonstrate a negative correlation with it (Figure 2b). The heatmap displayed a favorable association (Figure 2c) and a connection with RAB39B (Figure 2d). Furthermore, the LinkInterpreter module was used to perform GO and KEGG enrichment analyses on coexpressed genes of RAB39B. According to the GO functional annotation, the coexpressed gene RAB39B primarily participates in the replication, transportation, and synthesis of DNA and proteins (Figure 3a–c). According to the KEGG functional annotation, RAB39B and its associated genes primarily participate in the Wnt signaling pathway, Fanconi anemia pathway, T-cell receptor signaling pathway, and phosphatidylinositol signaling pathway. Additionally, they play a role in the synthesis and regulation of nucleotides and proteins. Furthermore, these genes also play a role in controlling the metabolic pathways of AML, including amino acid metabolism, carbon metabolism, nitrogen metabolism, phospholipid metabolism, and other biochemical processes (Figure 3d). These findings indicate that RAB39B may play a significant biological function in the development of AML.

### 3.3 Analysis of RAB39B in AML using immunofluorescence

**Figure 4:**
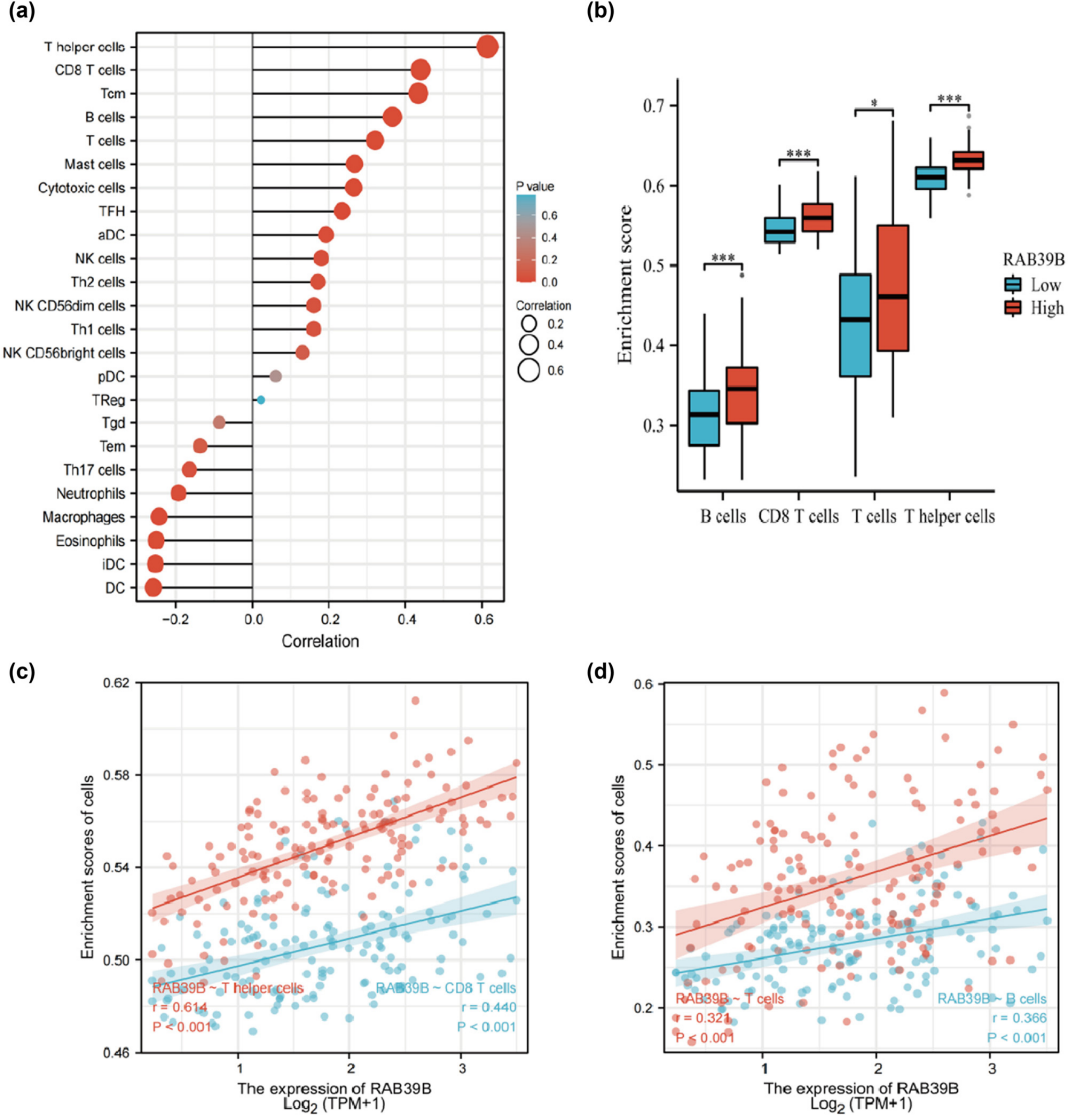
The correlation between RAB39B and immune markers in AML. (a) Correlation between the expression of RAB39B and 24 different types of immune cells. (b) Scatter plots depicting the correlation between RAB39B expression and the level of immune infiltration by T cells, B cells, T helper cells, and CD8 T cells. (c, d) The presence of T helper cells, T cells, CD8 T cells, and B cells is observed in cases with both low and high expression levels of RAB39B.

For the correlation analysis of the association between AML and 24 distinct immune cells, we utilized data extracted from TCGA database and employed Spearman’s method to assess the connection with RAB39B expression. According to Figure 4a, RAB39B exhibited a strong positive correlation with T helper cells (*r* = 0.614), CD8 T cells (*r* = 0.440), B cells (*r* = 0.366), and T cells (*r* = 0.321), but showed a negative correlation with dendritic cells (DCs, *r* = −0.288). Figure 4b displays a notable distinction in the scatter plot depicting the relationship between the enrichment score of specific immune cells and the expression level of RAB39B. The surface morphology of T helper cells, T cells, CD8 T cells, and B cells showed a notable variation between low and high expression of RAB39B, as depicted in Figure 4c.

### 3.4 Building ceRNA networks associated with RAB39B in AML

**Figure 5:**
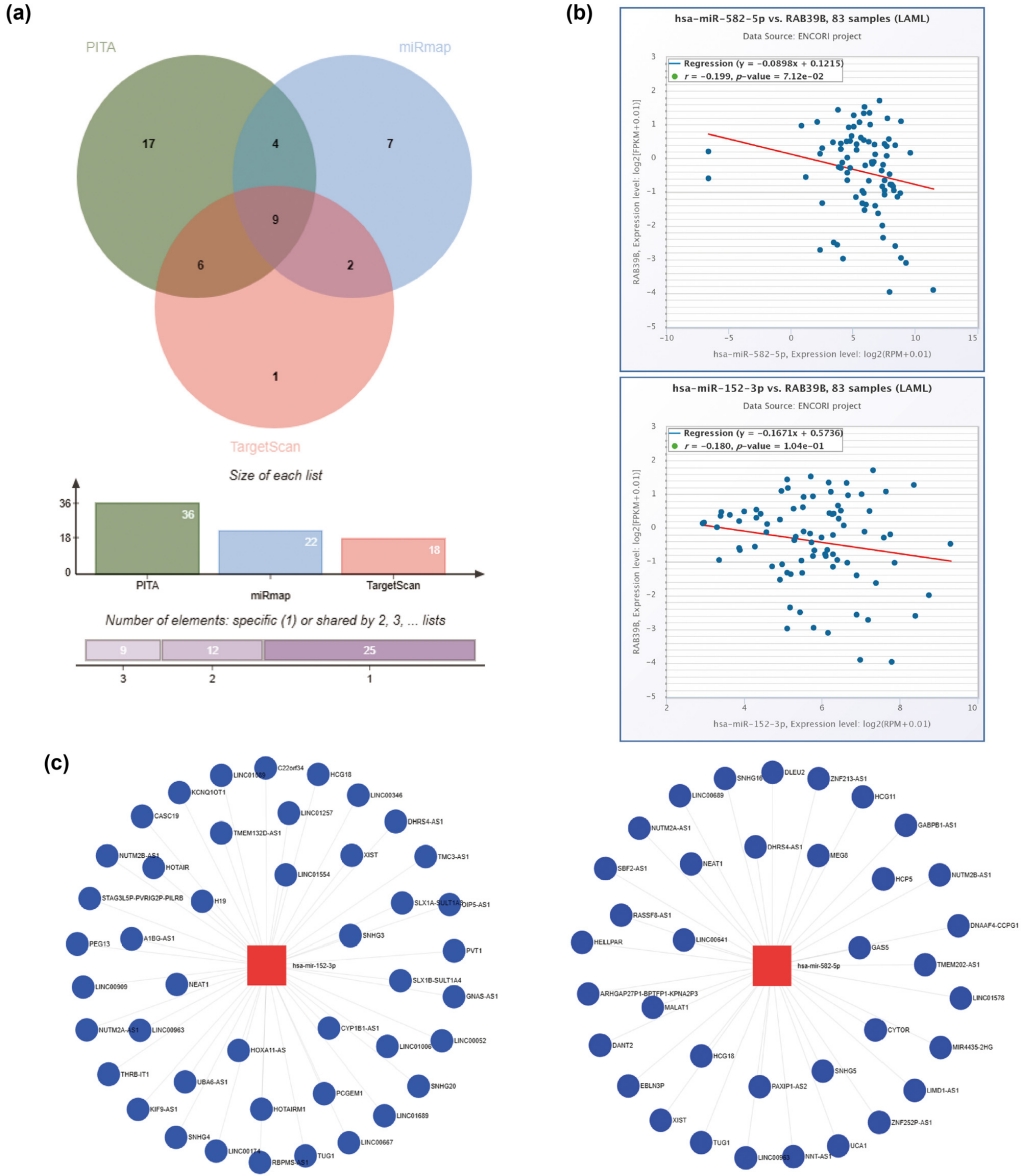

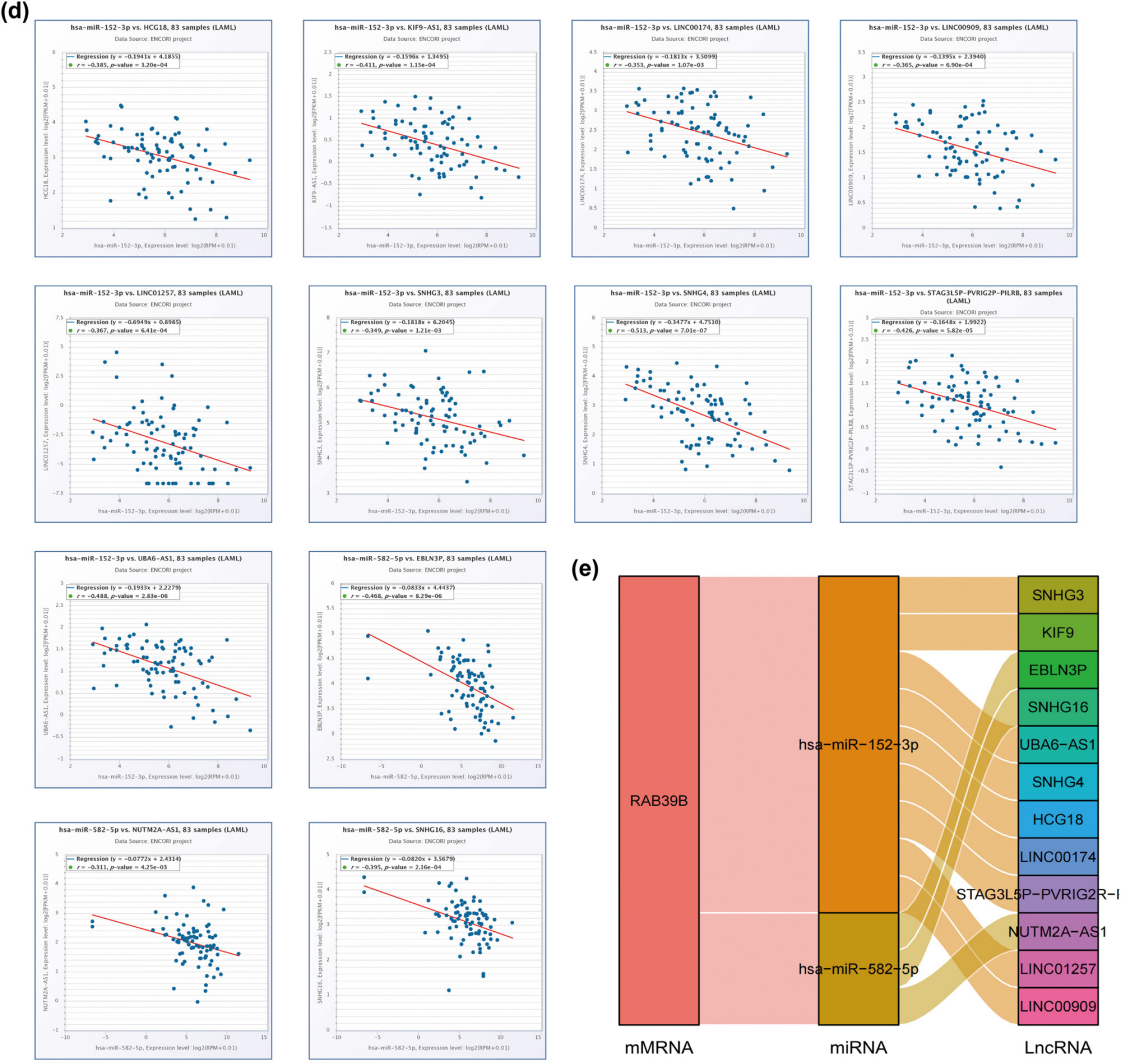
Construction of a ceRNA network associated with RAB39B in AML. (a) A Venn diagram illustrating the potential targets of RAB39B as predicted by PITA, miRmap, and TargetScan. (b) Scatter plots illustrating the correlation between RAB39B and the miRNAs of interest. (c) miRNet provides predicted target lncRNAs for hsa-miR-152-3p and hsa-miR-582-5p. (d) Scatter plots depict the correlation of hsa-miR-152-3p or hsa-miR-582-5p with the target lncRNAs. (e) The ceRNA hypothesis is supported by a sandwich figure illustrating the regulatory network of lncRNA miRNA mRNA (RAB39B).

The starBase database was utilized to screen the target miRANA of RAB39B in PITA, miRmap, and TargetScan. Thirty-six, 22, and 18 target miRNAs were obtained, respectively. From these three databases (Figure 5a), a sum of nine miRNAs that are commonly found was acquired. To construct ceRNAs, we screened miRNAs that were negatively correlated with RAB39B due to the regulation of target genes by miRNAs. In AML (Figure 5b), a noteworthy inverse association was discovered between hsa-miR-152-3p (*r* = −0.199, *p*-value = 7.12 × 10^−2^) and hsa-miR-582-5p (*r* = −0.180, *p*-value = 1.04 × 10^−1^), as well as the expression of RAB39B.

According to the ceRNA network hypothesis, a negative correlation was observed between lncRNAs and miRNAs (Figure 5c). A correlation analysis was performed, considering lncRNAs that exhibited a correlation coefficient lower than –0.30 with miRNA. Figure 5d shows that EBLN3P, NUTM2A-AS1, and SNHG16 exhibited a negative correlation with hsa-miR-582-5p, while SNHG3, KIF9, UBA6-AS1, SNHG4, HCG18, LINC00174, STAG3L5P-PVRIG2R-PILRB, LINC01257, and LINC00909 displayed a negative correlation with hsa-miR-152-3p. Using the Sangi Figure (Figure 5e), we created a total of 12 ceRNA networks.

### 3.5 The interaction between RAB39B and disconfidptosis in AML

**Figure 6:**
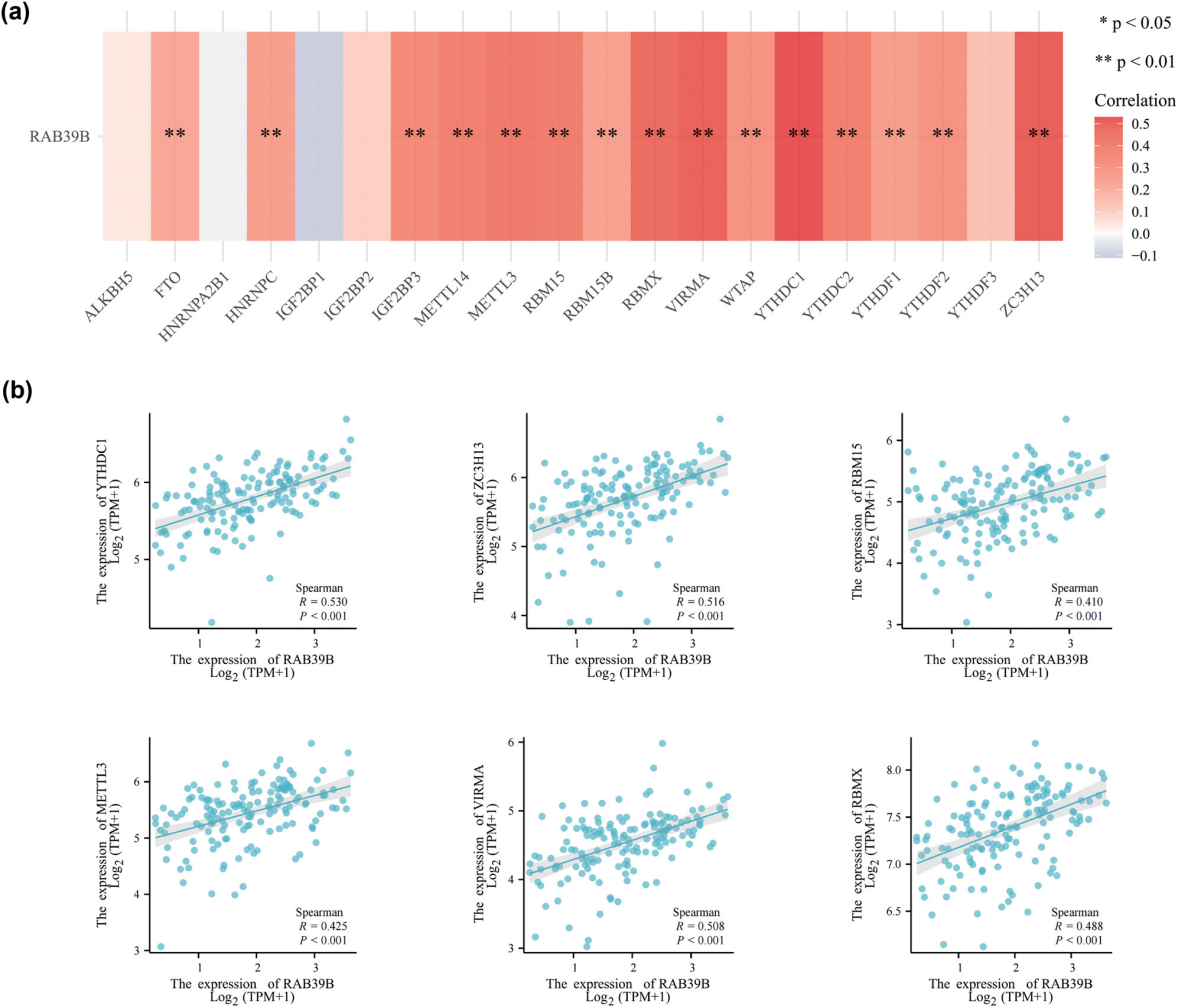
The association between RAB39B and m6A-associated genes in AML. (a) Examination of the association between RAB39B expression and the expression of genes related to m6A in TCGA AML cohort. (b) Scatter plots illustrating the relationship between RAB39B and genes associated with m6A.

To examine the correlation between RAB39B and m6A methylation, we explored the association between RAB39B and 20 m6A-associated genes in AML. A strong connection was discovered between RAB39B and 15 m6A-associated genes, namely, YTHDC1, YTHDC2, YTHDF1, YTHDF2, ZC3H13, WTAP, VIRMA, RBMX, RBM15B, RBM15, METTL3, METTL14, IGF2BP3, FTO, and HNRNPC (as shown in Figure 6a). Figure 6b presents scatter plots demonstrating the correlations between RAB39B and the top six most strongly correlated m6A-associated genes (YTHDC1, YTHDC2, YTHDF1, YTHDF2, ZC3H13, and WTAP), confirming the association of RAB39B with m6A methylation in AML.

### 3.6 Interaction between RAB39B and necroptosis in AML

**Figure 7:**
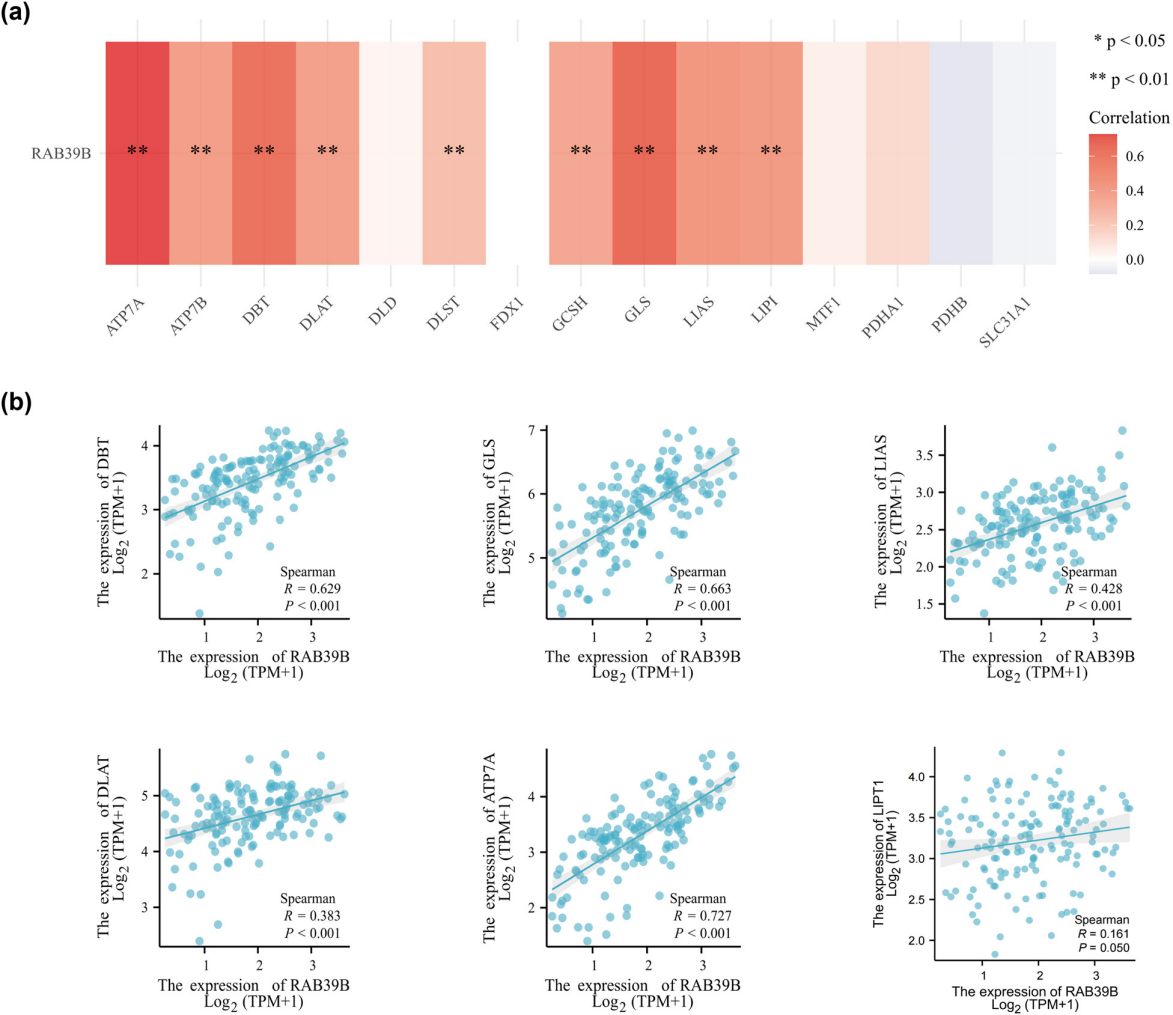
The relationship between RAB39B and genes associated with cuproptosis in AML. (a) Examining the association between the expression of RAB39B and genes related to cuproptosis in TCGA AML cohort. (b) Scatter plots illustrating the relationship between RAB39B and genes associated with cuproptosis.

Furthermore, we also investigated the correlation between RAB39B and cuprotosis. Out of the 15 genes associated with cuprotosis, 9 genes exhibited a notable and favorable relationship with the expression of RAB39B. These genes include ATP7A, ATP7B, DBT, DLAT, DLST, GCSH, GLS, LIAS, and LIPI (as shown in Figure 7a). The scatter plot of the top 6 correlations is depicted in Figure 7b. It suggests that RAB39B may play an important role in regulating cuprotosis-related processes.

### 3.7 Correlation between the expression of RAB39B and clinical characteristics and cytogenetic risk

The clinical characteristics of 150 AML patients (Table 1) retrieved from TCGA database demonstrate that RAB39B expression levels are significantly correlated with cytogenetic risk, FAB classifications (excluding the M3 type), bone marrow (BM) blast counts, and specific genetic mutations in FLT3 and NPM1. To be more specific, AML patients with high expression of RAB39B exhibited a significant association with adverse cytogenetic risk (*P* < 0.001) and specific FAB classifications (*P* < 0.001), excluding the M3 type due to its distinct treatment regimen involving ATRA. Additionally, high RAB39B expression was correlated with higher BM blast counts (*P* = 0.012) and showed a trend toward correlation with higher peripheral blood (PB) blast counts (*P* = 0.072) and white blood cell (WBC) counts (*P* = 0.060). Notably, patients with FLT3 mutations had lower expression of RAB39B (*P* = 0.026), while those with NPM1 mutations also tended to have lower RAB39B expression (*P* = 0.012). However, age, gender, race, WBC counts, PB blast counts, and other mutations (IDH1 R132, IDH1 R140, IDH1 R172, and RAS) did not show significant associations with RAB39B expression.

**Table 1:** Correlation between the expression of RAB39B and clinicopathological characteristics in AML samples retrieved from TCGA database

Characteristics	Low expression of RAB39B	High expression of RAB39B	*P* value
*n*	75	75	
**Gender,** ***n*** **(%)**			0.412
Female	36 (24%)	31 (20.7%)	
Male	39 (26%)	44 (29.3%)	
**Race,** ***n*** **(%)**			0.593
Asian and Black or African American	8 (5.4%)	6 (4%)	
White	67 (45%)	68 (45.6%)	
**Age,** ***n*** **(%)**			0.869
≤60	43 (28.7%)	44 (29.3%)	
>60	32 (21.3%)	31 (20.7%)	
**IDH1 R132 mutation,** ***n*** **(%)**			0.412
Negative	68 (45.9%)	67 (45.3%)	
Positive	5 (3.4%)	8 (5.4%)	
**IDH1 R140 mutation,** ***n*** **(%)**			0.961
Negative	69 (46.6%)	67 (45.3%)	
Positive	6 (4.1%)	6 (4.1%)	
**IDH1 R172 mutation,** ***n*** **(%)**			1.000
Negative	74 (50%)	72 (48.6%)	
Positive	1 (0.7%)	1 (0.7%)	
**FLT3 mutation,** ***n*** **(%)**			0.026
Negative	45 (30.8%)	56 (38.4%)	
Positive	29 (19.9%)	16 (11%)	
**RAS mutation,** ***n*** **(%)**			0.731
Negative	70 (47%)	71 (47.7%)	
Positive	5 (3.4%)	3 (2%)	
**NPM1 mutation,** ***n*** **(%)**			0.012
Negative	52 (34.9%)	64 (43%)	
Positive	23 (15.4%)	10 (6.7%)	
**BM blasts (%),** ***n*** **(%)**			0.012
≤20	37 (24.7%)	22 (14.7%)	
>20	38 (25.3%)	53 (35.3%)	
**PB blasts (%),** ***n*** **(%)**			0.072
≤70	30 (20%)	41 (27.3%)	
>70	45 (30%)	34 (22.7%)	
**WBC count (× 10** ^**9/L**^), ***n*** **(%)**			0.060
≤20	32 (21.5%)	44 (29.5%)	
>20	42 (28.2%)	31 (20.8%)	
**Cytogenetic risk,** ***n*** **(%)**			<0.001
Favorable	25 (16.9%)	5 (3.4%)	
Intermediate/normal	38 (25.7%)	44 (29.7%)	
Poor	11 (7.4%)	25 (16.9%)	
**FAB classifications,** ***n*** **(%)**			<0.001
M0	1 (0.7%)	14 (10.4%)	
M1	12 (8.9%)	23 (17.1%)	
M2	13 (9.6%)	25 (18.5%)	
M4	21 (15.6%)	8 (5.9%)	
M5	14 (10.4%)	1 (0.7%)	
M6	0 (0%)	2 (1.5%)	
M7	0 (0%)	1 (0.7%)	

To further confirm the connection between AML clinical factors and RAB39B (Table 2), logistic regression analysis was employed. RAB39B expression showed a positive correlation with cytogenetic risk (*P* = 0.009) and BM blasts (*P* = 0.013) but exhibited a negative correlation with FLT3 mutation (*P* = 0.028) and NPM1 mutation (*P* = 0.014).

**Table 2:** The relationship between the clinicopathological factors of AML and RAB39B expression by using logistic regression analysis

Characteristics	Total (*N*)	OR (95% CI)	*P* value
Age (>60 vs ≤60)	150	0.947 (0.298–1.595)	0.869
WBC count (×10^9/L^) (>20 vs ≤20)	149	0.537 (–0.113–1.187)	0.061
Cytogenetic risk (poor vs favorable and intermediate/normal)	148	2.922 (2.121–3.724)	0.009
BM blasts (%) (>20 vs ≤20)	150	2.346 (1.673–3.018)	0.013
PB blasts (%) (>70 vs ≤70)	150	0.553 (–0.095–1.201)	0.073
FLT3 mutation (positive vs negative)	146	0.443 (–0.282–1.169)	0.028
IDH1 R132 mutation (positive vs negative)	148	1.624 (0.457–2.791)	0.416
IDH1 R140 mutation (positive vs negative)	148	1.030 (–0.151–2.210)	0.961
IDH1 R172 mutation (positive vs negative)	148	1.028 (–1.763–3.819)	0.985
NPM1 mutation (positive vs negative)	149	0.353 (–0.474–1.181)	0.014

The OS of AML patients was influenced by RAB39B (high vs low, *P* = 0.028), cytogenetic risk (favorable and intermediate/normal vs poor, *P* < 0.019), and age (≤60 vs >60, *P* < 0.001) in the univariate Cox model (Table 3). Age (≤60 vs >60, *P* < 0.001) emerged as the sole independent variable linked to OS in patients with AML, as per the multivariate Cox model.

**Table 3:** Univariate and multivariate Cox regression analyses of factors associated with OS in AML [36,37]

Characteristics	Total (*N*)	Univariate analysis		Multivariate analysis	
		Hazard ratio (95% CI)	*P* value	Hazard ratio (95% CI)	*P* value
Age	139				
≤60	78	Reference		Reference	
>60	61	3.321 (2.156–5.116)	<0.001	3.193 (2.042–4.992)	<0.001
Cytogenetic risk	137				
Favorable and intermediate/normal	106	Reference		Reference	
Poor	31	1.803 (1.102–2.951)	0.019	1.356 (0.820–2.243)	0.235
Gender	139				
Female	62	Reference			
Male	77	1.024 (0.671–1.564)	0.912		
Race	138				
Asian and Black or African American	11	Reference			
White	127	1.200 (0.485–2.966)	0.693		
WBC count (×10^9/L^)	138				
≤20	74	Reference			
>20	64	1.156 (0.757–1.764)	0.503		
BM blasts (%)	139				
≤20	58	Reference			
>20	81	1.159 (0.754–1.780)	0.502		
PB blasts (%)	139				
≤70	65	Reference			
>70	74	1.224 (0.802–1.869)	0.349		
FLT3 mutation	135				
Negative	96	Reference			
Positive	39	1.266 (0.798–2.009)	0.316		
IDH1 R132 mutation	137				
Negative	125	Reference			
Positive	12	0.586 (0.237–1.448)	0.247		
IDH1 R140 mutation	137				
Negative	126	Reference			
Positive	11	1.129 (0.564–2.258)	0.733		
IDH1 R172 mutation	137				
Negative	135	Reference			
Positive	2	0.608 (0.085–4.376)	0.621		
NPM1 mutation	138				
Negative	105	Reference			
Positive	33	1.134 (0.704–1.826)	0.606		
RAS mutation	138				
Negative	130	Reference			
Positive	8	0.642 (0.235–1.756)	0.388		
RAB39B	71				
Low	38	Reference		Reference	
High	33	1.980 (1.076–3.641)	0.028	1.520 (0.776–3.041)	0.274

### 3.8 Prognostic correlation analysis of RAB39B in AML

**Figure 8:**
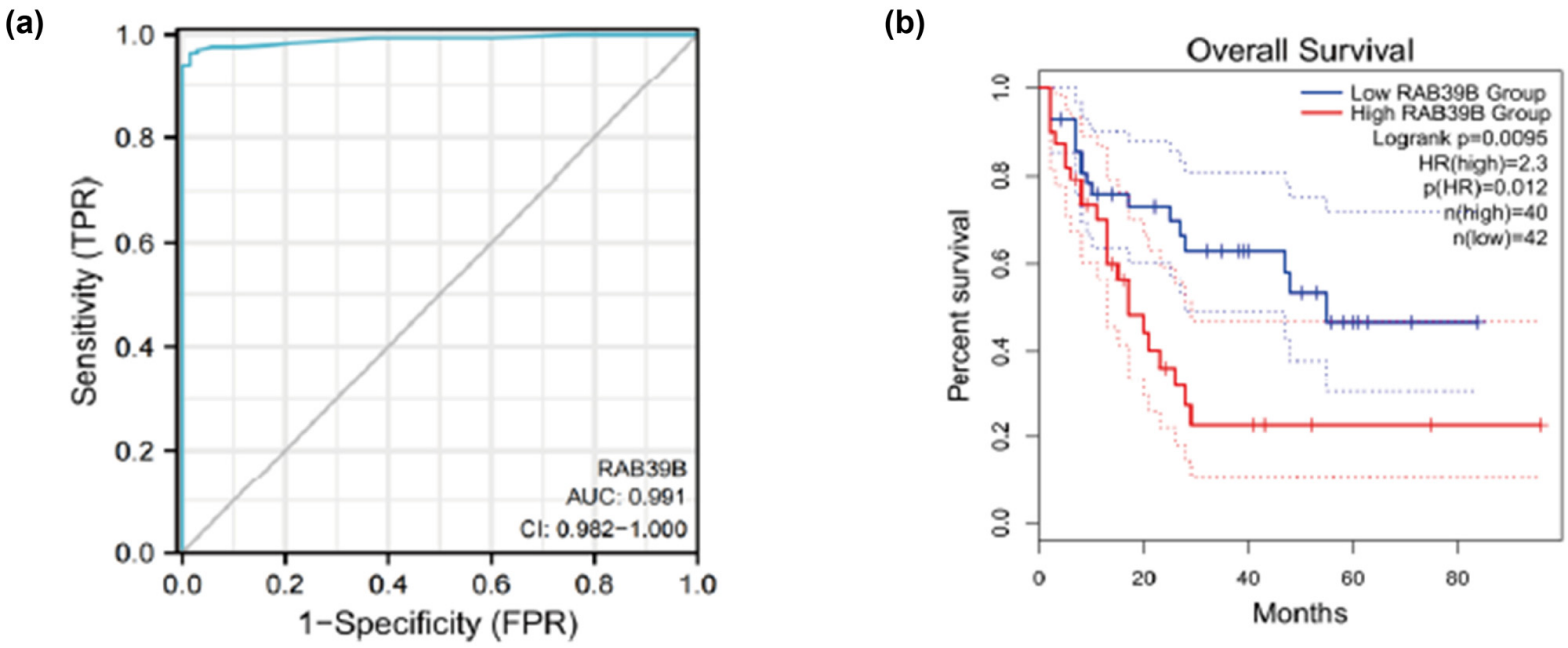
(a) Draw ROC curve chart based on prognostic analysis. (b) Draw a Kaplan Meier curve based on prognostic analysis.

Through ROC curve analysis of data from UCSC XENA, we investigated the ability of RAB39B to distinguish between healthy cells and AML cells. The result (Figure 8a) revealed an area under the curve of 0.991, highlighting its high sensitivity and specificity in identifying AML. Furthermore, utilizing the GEPIA2 database to examine the Kaplan‒Meier survival plot, it was observed that an elevated level of RAB39B expression was linked to an unfavorable prognosis (HR = 2.3, *P* < 0.05) (Figure 8b).

### 3.9 The prognostic model of *RAB39B* in AML

**Figure 9:**
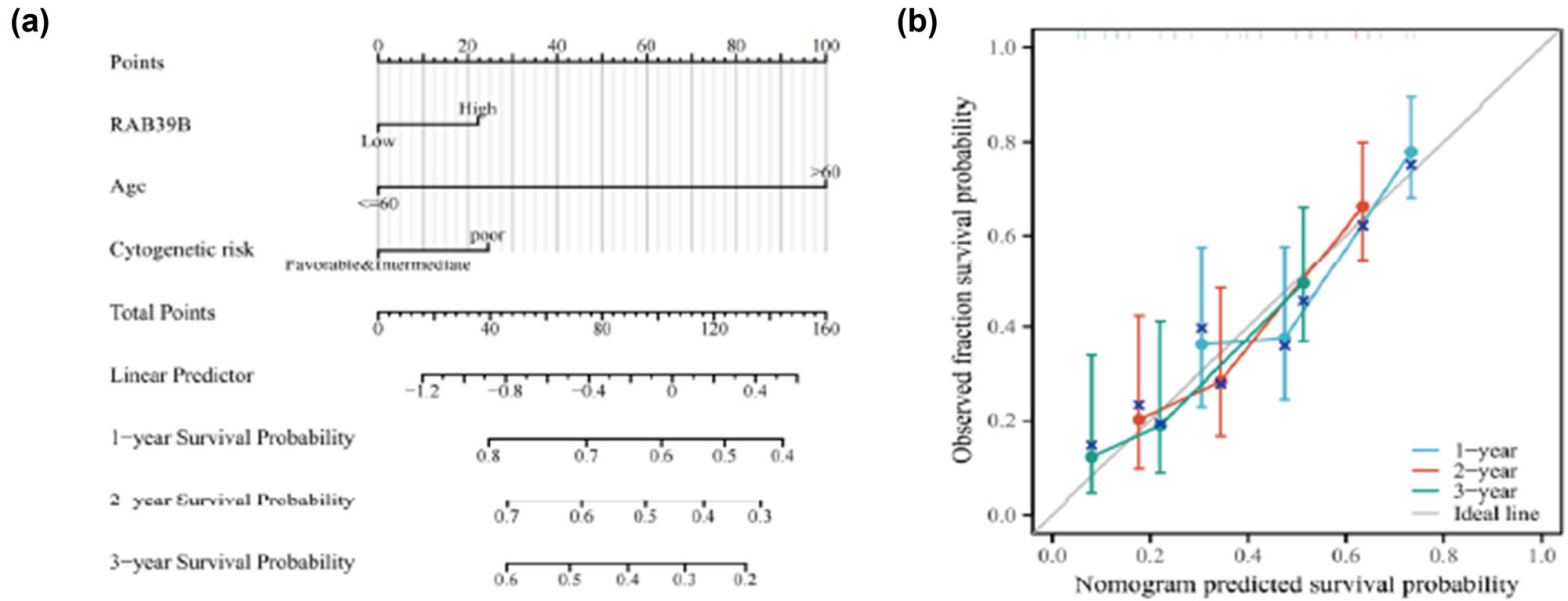
An AML prognostic predictive model for RAB39B. (a) A nomogram is available to estimate the likelihood of surviving for 1, 2, and 3 years for AML. (b) A calibration plot of the nomogram for predicting OS at 1, 2, and 3 years.

Based on the results of the previous multivariate Cox regression analysis, we identified three independent prognostic factors: age, cytogenetic risk, and RAB39B expression. Utilizing these factors, we constructed an AML prognostic prediction nomogram model (Figure 9a) to estimate the 1-, 2-, and 3-year survival rates of AML patients. In Figure 9b, the calibration curves for 1-, 2-, and 3-year survival rates all closely approximate the 45° line, indicating that this prognostic prediction model exhibits high accuracy in predicting the survival rates of AML patients.

### 3.10 *In vitro* validation of RAB39B’s function in AML

**Figure 10:**
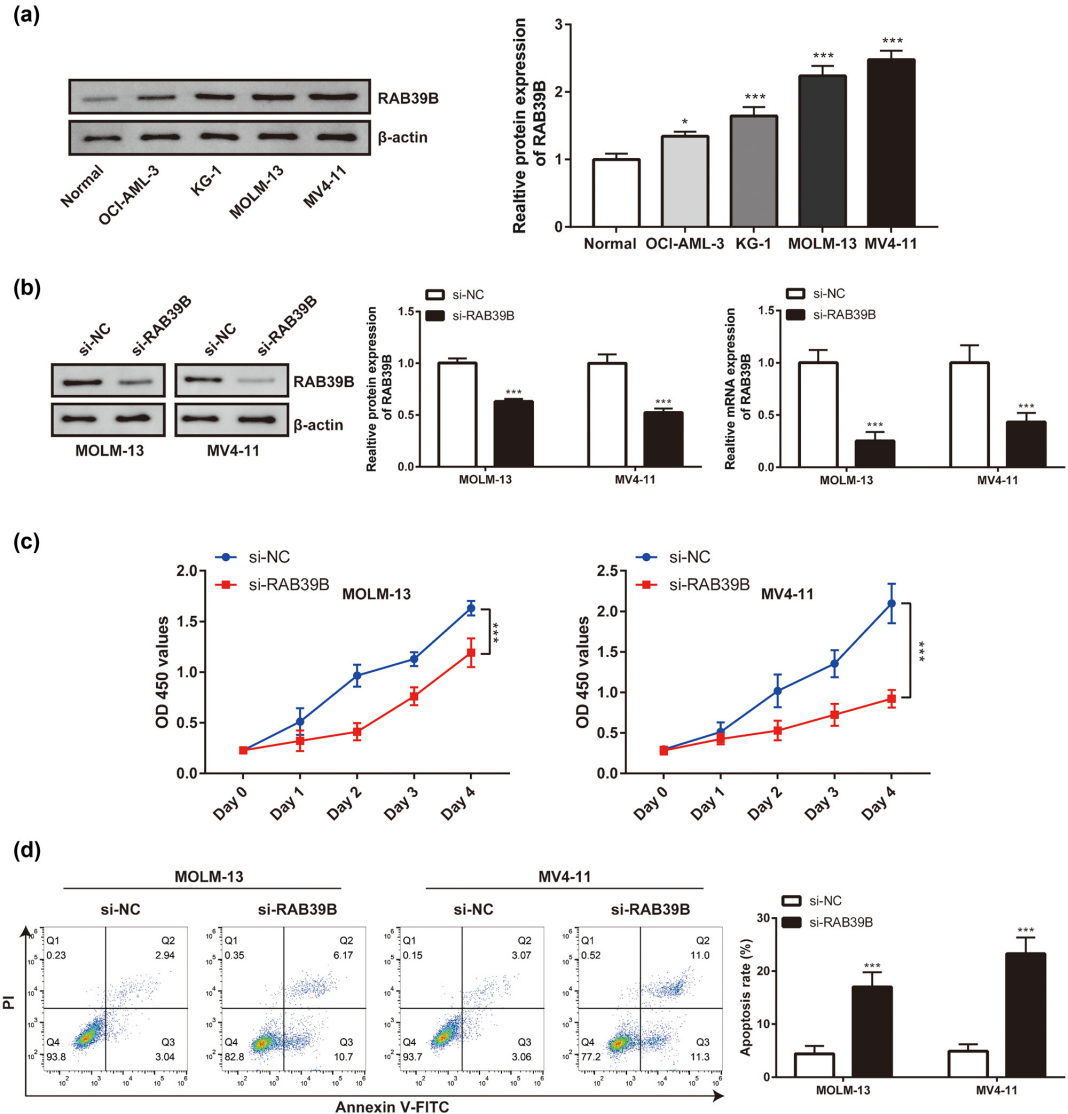
AML is linked to the expression and proliferation of RAB39B in cells. (a) The expression of RAB39B in various cell lines was detected using Western blotting and qPCR. (b) qPCR and Western blotting techniques were used to measure the transfection effectiveness of RAB39B siRNA in MOML-13 and MV4-11 cells. (c) The CCK-8 proliferation experiment revealed that suppressing RAB39B can result in reduced AML cell proliferation. (d) Knocking down RAB39B leads to increased apoptosis in AML cells.

Initially, Western blot analysis was employed to identify the contrasting expression of RAB39B in various cellular lineages (OCI-AML-3, KG-1, MOLM-13, and MV4-11 cell lines). The expression of RAB39B was higher in all four of these AML cell lines than in regular peripheral blood monocytes (Figure 10a). Given that the MOML-13 and MV4-11 cell lines exhibited relatively significant differences in RAB39B expression in the Western blot analysis, we therefore focused our subsequent research on these two representative cell lines. Through siRNA transfection (Figure 10b), the expression of RAB39B was inhibited in the MOML-13 and MV4-11 cell lines. In the CCK-8 study, it was observed that the growth of AML cell lines declined following the suppression of RAB39B (as depicted in Figure 10c). Conversely, the suppression of RAB39B also resulted in an elevation of AML cell apoptosis (as illustrated in Figure 10d).

## 4 Discussion

AML is the prevailing form of acute leukemia among adults. Despite thorough investigation to discover predictive biomarkers, AML continues to be a condition characterized by notable prognostic variations and elevated fatality rates. Rab proteins, which are small GTPases, play a role in controlling the movement of vesicles within eukaryotic cells. Rab39B is a member of the Rab protein family and can be found on the Xq28 region of the human chromosome. Rab proteins, which play a significant role in controlling vesicle trafficking, have emerged as crucial regulators of various cellular processes, including cell growth, differentiation, survival, autophagy, and apoptosis [14,15,20,21,38]. Rab proteins impact membrane trafficking and trafficking complexes, thereby regulating cell metabolism, apoptosis, and autophagy. They also have a significant role in tumors, neurological diseases, and other genetic diseases, ultimately influencing multiple signaling pathways.

Several RAB genes have been discovered to have a connection with tumors. Various RAB genes have been linked to tumors, including RAB27A [39], which serves as a predictive indicator for pancreatic ductal adenocarcinoma; RAB13 and RAB3D, which are elevated in pan-cancer [40,41]; and RAB1A and RAB6A, which have significant functions in rectal cancer and bile duct cancer [42,43], respectively. Numerous atypical Rab proteins are believed to be linked to malignant tumors. Research indicates that RAB39B exhibits increased expression in various types of tumors, such as germ cell tumors, gastric stromal tumors, and diffuse large B-cell lymphoma [16,17,18]. Moreover, the removal and excessive expression of RAB39B impact cellular autophagic flow [35,44]. Nevertheless, the current lack of comprehensive research on the mechanism and prognostic significance of RAB39B in AML remains evident.

Through analysis of TCGA data, we found that RAB39B has significant expression differences in most cancers and AMLs of pan-cancer. *In vitro* experiments confirmed this as well. By conducting PPI and coexpression gene enrichment analysis, we discovered that RAB39B is probably engaged in substance conveyance, DNA replication and conveyance, substance metabolism, and more within AML cells. Consequently, it governs the functions of apoptosis and autophagy in AML. Furthermore, numerous pathways associated with tumors were identified, including the Wnt signaling pathway, T-cell receptor signaling pathway, and phosphatidylinositol signaling pathway. Notably, the Fanconi anemia pathway, which plays a crucial role in DNA damage repair, was found to interact with multiple pathways and contribute to the progression of AML [45]. By analyzing these data, we can gain insight into the possible roles of RAB39B in the onset and progression of AML.

The presence of immune cells in the tumor microenvironment is a crucial factor, as studies have indicated that it plays a significant role in both tumor progression and response to treatment. The cellular foundation of antitumor immunity is primarily composed of tumor-infiltrating immune cells, particularly T cells. Additionally, studies have demonstrated the significance of various immune cells, such as DCs, B lymphocytes, and natural killer cells. B lymphocytes and dendritic cells play a multifaceted role in the growth of tumors and the surrounding environment, serving as both catalysts for tumor advancement and, conversely, impediments to it [46]. Viewing AML from a different angle may shed light on the immune evasion mechanism, leading to the development of novel treatment approaches and a deeper comprehension of immune cell infiltration within the AML tumor microenvironment. This, in turn, can enhance our understanding of the role of RAB39B in AML. Analysis of immune infiltration revealed a strong positive correlation between RAB39B and T helper cells, T cells, CD8 T cells, and B cells but a negative correlation with DCs. The findings indicate that RAB39B might contribute to unfavorable outcomes in AML through its impact on immune infiltration. In addition, in the past few years, new targeted T-cell therapy methods for AML have been developing, including the construction of T-cell receptors, reactivation of endogenous T-cell responses through immune checkpoint inhibitors, and so on. At present, these immunotherapies are not yet complete and require new biomarkers and immune checkpoints to better regulate the tumor microenvironment and cell therapy. From this, we can foresee the potential of RAB39B in tumor immunotherapy [47].

Using miRNA shas-miR-152-3p and hsa-miR-582-5p, along with lncRNAs EBLN3P, NUTM2A-AS1, SNHG16, SNHG3, KIF9, UBA6-AS1, SNHG4, HCG18, LINC00174, STAG3L5P-PVRIG2R-PILRB, LINC01257, and LINC00909, we established a network consisting of lncRNA‒miRNA‒mRNA (RAB39B). Among them, EBLN3P, SNHG proteins and HCG18 [48,49,50,51] have been identified as driving factors for various tumors. At present, we have only constructed a credible network model, and further experiments are needed to verify this finding.

The study on m6A methylation and AML revealed that YTHDF1, METTL14, YTHDC2, ZC3H13, RBM15, WTAP, METTL3, METTL14, FTO, IGF2BP3, and ALKBH5 are among the m6A genes implicated in the development of AML. The heatmap indicates that all the aforementioned genes, excluding ALKBH5, exhibit a strong correlation with RAB39B in AML. This finding enhances our comprehension of the mechanism underlying RAB39B in AML [52,53,54,55,56,57,58,59]. The literature states that AML has three cuproptosis genes, namely, GCSH, LIPT1, and DLAT [60,61], which possess individual and noteworthy prognostic significance. All three genetic factors in our research exhibited a strong correlation with RAB39B. The presence of these occurrences indicates a connection between m6A methylation and cuproptosis in AML, suggesting that RAB39B could exert a significant influence on these vital biological mechanisms. We should conduct further experiments to continue to explore this issue.

To validate the correlation between RAB39B and the survival rate of AML patients, we employed ROC curves and Kaplan‒Meier survival analysis. The ROC curve shows that RAB39B can distinguish between tumor cells and normal cells, and the expression of RAB39B is significantly correlated with the survival rate of AML patients. Through logistic, univariate, and multivariate Cox analyses, we additionally assessed the expression of RAB39B and other clinical-pathological factors associated with OS in AML. We can observe that the expression of RAB39B is associated with NPM1 and FLT3 mutations. Studies have found that AML associated with these two mutations generally has a good prognosis, and this relationship is worth further exploration to seek new treatment options through rab protein on the basis of specific mutations [62]. RAB39B, cytogenetic risk, and age can all be considered separate predictors in the univariate Cox analysis. Incorporating RAB39B, cytogenetic risk, and age into the construction of prognostic column charts and calibration charts, the calibration charts showed consistent results with RAB39B column charts for 1, 2, and 3 years. The findings suggest that an elevated level of RAB39B is linked to an unfavorable prognosis, making it a reliable marker for prognosticating AML outcomes.

Ultimately, we performed an *in vitro* confirmation of the initial analysis, and the findings indicated a variation in RAB39B expression between AML cell lines and typical human peripheral blood monocytes. By suppressing the expression of RAB39B, we observed a reduction in AML cell growth and an elevation in cell death. The preliminary stage of the *in vitro* experiment ensured the functional analysis of RAB39B in AML.

## 5 Conclusions

The objective of our study was to investigate the function and predictive significance of RAB39B in AML. We discovered an upsurge in RAB39B expression in AML for the first time. Additionally, we determined the potential pathways and mechanisms through which RAB39B contributes to the development of AML. Furthermore, we conducted a novel analysis of the correlation between RAB39B expression and AML immune infiltration, m6A modification, copper-induced cell death, ceRNA network, and prognosis. We also conducted concise validation using *in vitro* experiments. Currently, we believe that RAB39B has the potential to function as both a diagnostic and prognostic indicator for AML.

## Abbreviations

AML: Acute myeloid leukemia
TCGA: The Cancer Genome Atlas
GTEx: Genotype-tissue expression
DEGs: Differentially expressed genes
GSEA: Gene set enrichment analysis
GO: Gene Ontology
KEGG: Kyoto Encyclopedia of Genes and Genomes
STRING: Search Tool for the Retrieval of Interacting Genes
PPI: Protein–protein interaction
ROC: Receiver operating characteristic
